# Characterization of Ravn virus viral shedding dynamics in experimentally infected Egyptian rousette bats (*Rousettus aegypticus*)

**DOI:** 10.1128/jvi.00045-25

**Published:** 2025-04-23

**Authors:** Jessica A. Elbert, Amy J. Schuh, Brian R. Amman, Jonathan C. Guito, James C. Graziano, Tara K. Sealy, Elizabeth W. Howerth, Jonathan S. Towner

**Affiliations:** 1 Department of Pathology, College of Veterinary Medicine, University of Georgia 70734, Athens, Georgia, USA; 2 Viral Special Pathogens Branch, Division of High-Consequence Pathogens and Pathology, National Center for Emerging and Zoonotic Infectious Diseases, United States Centers for Disease Control and Prevention 164542, Atlanta, Georgia, USA; 3 USA Public Health Service Commissioned Corps 33426 , Rockville, Maryland, USA; The Ohio State University, Columbus, Ohio, USA

**Keywords:** Ravn virus, Marburg virus, Egyptian rousette bat, filovirus, bat

## Abstract

Marburg virus (MARV) and Ravn virus (RAVV), the only two known members of the species *Orthomarburgvirus marburgense* (family *Filoviridae*), are causative agents of Marburg virus disease, a severe viral disease that typically emerges in sub-Saharan Africa and is characterized by human-to-human transmission and high case fatalities. Despite the robust characterization of MARV experimental infection in Egyptian rousette bats (ERBs; *Rousettus aegyptiacus*; common name: Egyptian rousettes), a natural MARV reservoir, experimental infection with RAVV in ERBs has not been completed. Here, we experimentally infect 12 ERBs with RAVV and quantify viral loads in blood, oral swabs, and rectal swabs over a 21-day timeline with serological and cumulative shedding data and baseline clinical parameters. Compared to previously described experimental MARV infection in ERBs, these bats experimentally inoculated with RAVV had significantly higher and prolonged rectal viral shedding loads, as well as significantly prolonged oral shedding and higher peak viremia. All ERBs seroconverted by 21 days post-infection. Additionally, all ERBs demonstrated marked heterogeneity in RAVV viral shedding loads consistent with the Pareto Principle and viral “supershedders.” Our results introduce the possibility of variation in transmission dynamics and subsequent spillover differences between RAVV and MARV.

**IMPORTANCE:**

Ravn virus, along with Marburg virus, causes severe viral disease in humans with high fatality but little to no clinical disease in its reservoir host, the Egyptian rousette bat. Our findings provide important insights into how Ravn virus behaves in its natural reservoir host, showing that Ravn virus infection followed a similar timeline to Marburg virus infection, with virus detected in blood, saliva, and feces. However, Ravn virus-infected bats had higher levels of viral shedding and shed the virus for a longer period, particularly in feces, compared to Marburg virus. These differences in viral shedding may impact the spread of the virus within bat populations and potentially alter the likelihood of spillover into humans, non-human primates, and other animal species. These insights are crucial for understanding Ravn virus maintenance in its bat reservoir and improving our ability to mitigate or prevent future human outbreaks.

## INTRODUCTION

Marburg virus (MARV) and Ravn virus (RAVV), viral relatives to Ebola virus, are the only two known members of the species *Orthomarburgvirus marburgense* (family *Filoviridae*, genus *Orthomarburgvirus*) and are the causative agents of Marburg virus disease (MVD), a severe and often fatal disease that typically emerges in sub-Saharan Africa characterized by human-to-human transmission and high case fatality ratios up to 90% (1). MARV was first identified in 1967 after laboratory workers in Marburg and Frankfurt, Germany and Belgrade (former Yugoslavia) became ill after working with African green monkeys imported from Uganda (2). To date, there have been 18 known MARV outbreaks, the most recent of which occurred in Tanzania in January 2025 (3). RAVV was first identified in 1987 following a fatal VHF case in a tourist who visited Kitum Cave in Mount Elgon National Park, Kenya (4). Designated as a distinct virus within the *Orthomarburgvirus marburgense* species in 1996, RAVV has since been identified in two subsequent outbreaks, with the most recent case reported in Uganda in 2007 (4–6). Although details about its natural distribution are limited, RAVV has only been detected in regions of Africa where Egyptian rousette bats (*Rousettus aegyptiacus,* common name: Egyptian rousettes) are found (7, 8).

The Egyptian rousette bat (ERB) is a pteropodid bat (order Chiroptera, family Pteropodidae) that inhabits parts of Africa, western Asia, the Mediterranean, and the Indian subcontinent (9). One of the few pteropodid bats capable of echolocation, they are predominantly cave-dwelling, gregarious, and social animals living in densely packed roosts (9). Numerous longitudinal ecological studies have identified the ERB as a natural reservoir host for both RAVV and MARV (6, 8, 10–13). These studies revealed that adult ERBs had the highest IgG antibody levels, while juvenile bats (approximately 6 months old) showed the highest levels of active infection, along with a temporal association between MARV disease spillover to humans and seasonal, biannual pulses of active MARV infection in juvenile ERBs (6, 10, 14). MARV and RAVV have been isolated multiple times from ERBs sampled in Uganda (6, 10, 15).

**Fig 1:**
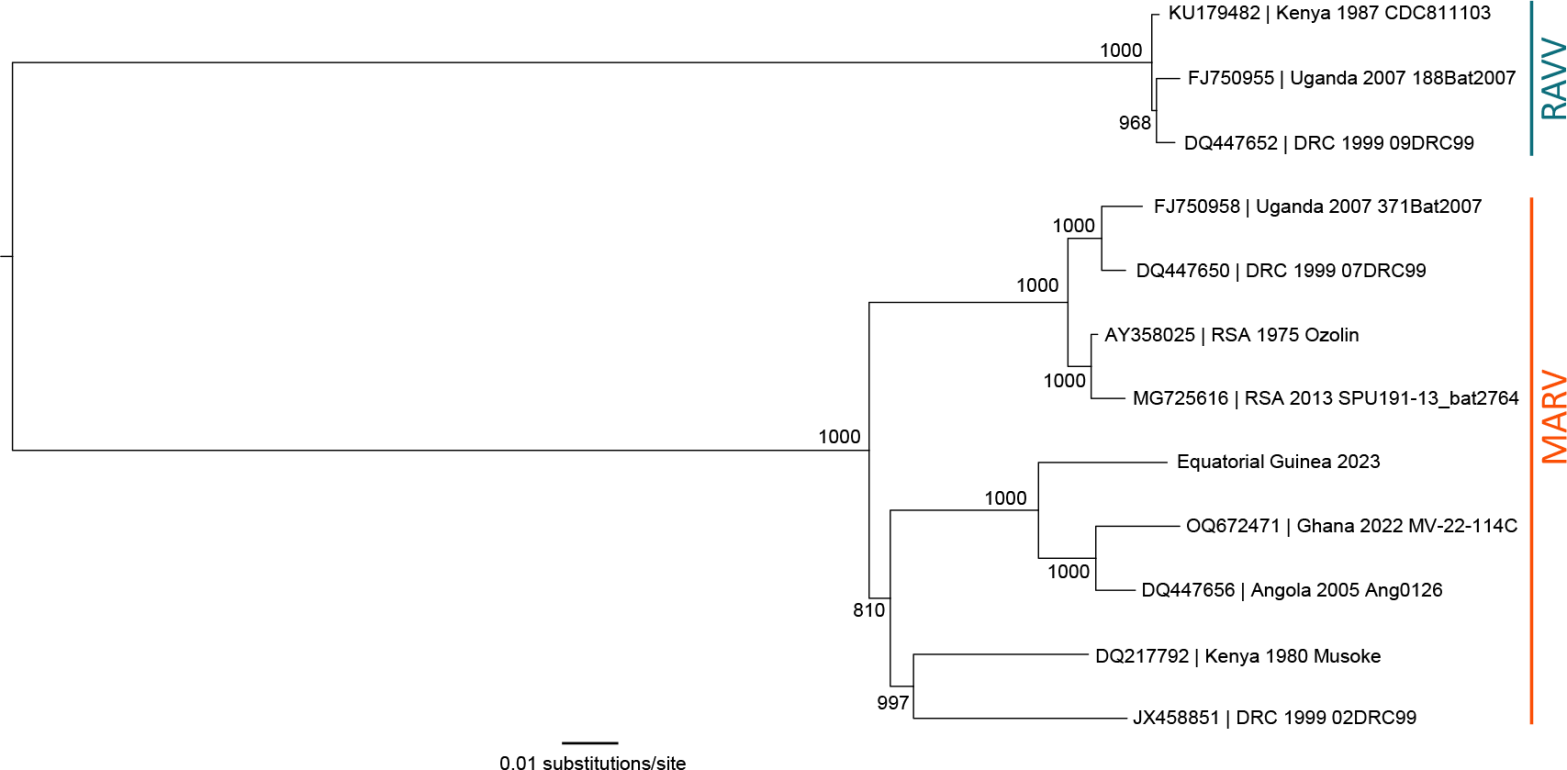
Midpoint-rooted, maximum-likelihood phylogeny of a subset of complete Marburg virus and Ravn virus genomes. Complete sequences from GenBank (accession numbers indicated) were aligned using Geneious Prime version 2024.0 (https://www.geneious.com/). The Equatorial Guinea 2023 sequence was acquired from https://virological.org/t/first-emergence-of-marburg-virus-in-equatorial-guinea-2023/924. ATGC Montpellier Bioinformatics Platform PhyML 3.0 (http://www.atgc-montpellier.fr/phyml/) was used to infer the maximum-likelihood tree after 1,000 bootstrap replicates. Node values indicate bootstrap support values. Scale bar indicates nucleotide substitutions per site. MARV, Marburg virus; RAVV, Ravn virus.

Despite being within the same viral species, the full genomic sequence of RAVV differs by up to 21% from MARV, and the amino acid sequence of the RAVV glycoprotein (GP) differs by ~22% from MARV GP (6, 16). Bayesian coalescent analysis has estimated that the most recent common ancestor (MRCA) between MARV and RAVV was approximately 700 years ago, with the known human and bat RAVV isolates sharing an MRCA approximately 50 years ago (17). A phylogenetic tree highlighting a subset of complete MARV and RAVV genomes is provided in Fig. 1.

Experimental studies have identified the ERB as a competent natural reservoir model for MARV (11, 18–21) and have also documented successful horizontal transmission of MARV between experimentally inoculated and naïve co-housed ERBs (14). ERBs experimentally inoculated with MARV have transient subclinical disease characterized by viremia (mean duration = 6.0 days, mean day of mean peak load = 6.8 DPI) (14), oral shedding (mean duration = 4.6 days, mean day of mean peak load = 9.1 DPI) with successful isolation of infectious MARV from 9/51 (17.6%) of MARV RT-qPCR-positive oral swabs (14), and rectal shedding (mean duration = 1.5 days, mean day of mean peak load = 6.8 DPI) (14). MARV shedding in the urine of experimentally infected ERBs has been documented but is limited by the challenging nature of the non-invasive specimen collection and is not fully characterized (14, 21). In previous studies, MARV-inoculated ERBs robustly seroconverted to MARV, with MARV IgG antibodies peaking between 12 and 28 DPI (14, 20, 21), followed by a decline of antibody levels falling below the threshold of seropositivity by 3 months post-infection (14). Despite diminished IgG levels, robust longstanding immunity to reinfection upon experimental challenge with MARV has been documented in ERBs up to 2 years after initial infection (22).

MARV has been well studied in numerous animal models and *in vitro* and *in vivo* experimental studies, including recent research utilizing transcriptomics, to elucidate ERB immunology and responses to infection (23–35). However, there has been limited experimental characterization of RAVV and of comparisons of virulence between RAVV and MARV. To date, the few studies utilizing RAVV include vaccine efficacy studies in cynomolgus macaques (36, 37), mice (38), and guinea pigs (39), therapeutic treatment trials of MARV and RAVV infection in non-human primates with human monoclonal antibodies (40) and small interfering RNA (41), and characterization of the lack of observable clinical disease upon experimental inoculation with RAVV in ferrets (42, 43). Genetic variation between MARV and RAVV could influence transmission dynamics, pathogenicity, and, potentially, responses to treatments or vaccines. A recent study comparing experimental infections of different orthomarburgviruses in macaques found distinct pathogenicities between RAVV, MARV Angola, and variant isolates Musoke and Ozolin, and additionally found that despite seroconversion in all animals, RAVV is lethal in cynomolgus macaques but not rhesus macaques (44). Furthermore, a comparison of the pathogenesis of RAVV, MARV variant isolates Musoke and Popp, and MARV Angola in a serially adapted outbred guinea pig model found delayed increases in circulating inflammatory and prothrombotic elements, lower viremia levels, less severe histologic disease, and a delay in mean time to death in RAVV infection compared to MARV Angola (27). To date, experimental characterization of RAVV infection in its ERB reservoir has not been completed.

Here, we present an initial characterization of viral infection and shedding dynamics in ERBs experimentally infected with RAVV, with an aim to elucidate differences between RAVV and MARV and establish parameters for RAVV experimental infection in ERBs. We performed a 22-day experiment using 12 captive-bred and age- and sex-matched ERBs subcutaneously inoculated with a low-passage (P2) wild-type RAVV (188Bat2007) isolated from a naturally infected bat in Uganda (6). This work provides an important baseline for hypothesis-driven research, allowing a successful extrapolation of research findings in controlled laboratory settings to wild ERB populations and enabling experimental comparisons between MARV and RAVV infections. Additionally, this work furthers the continued validation of the only established reservoir model for any filovirus.

## RESULTS

### RAVV replication and shedding dynamics

**Fig 2:**
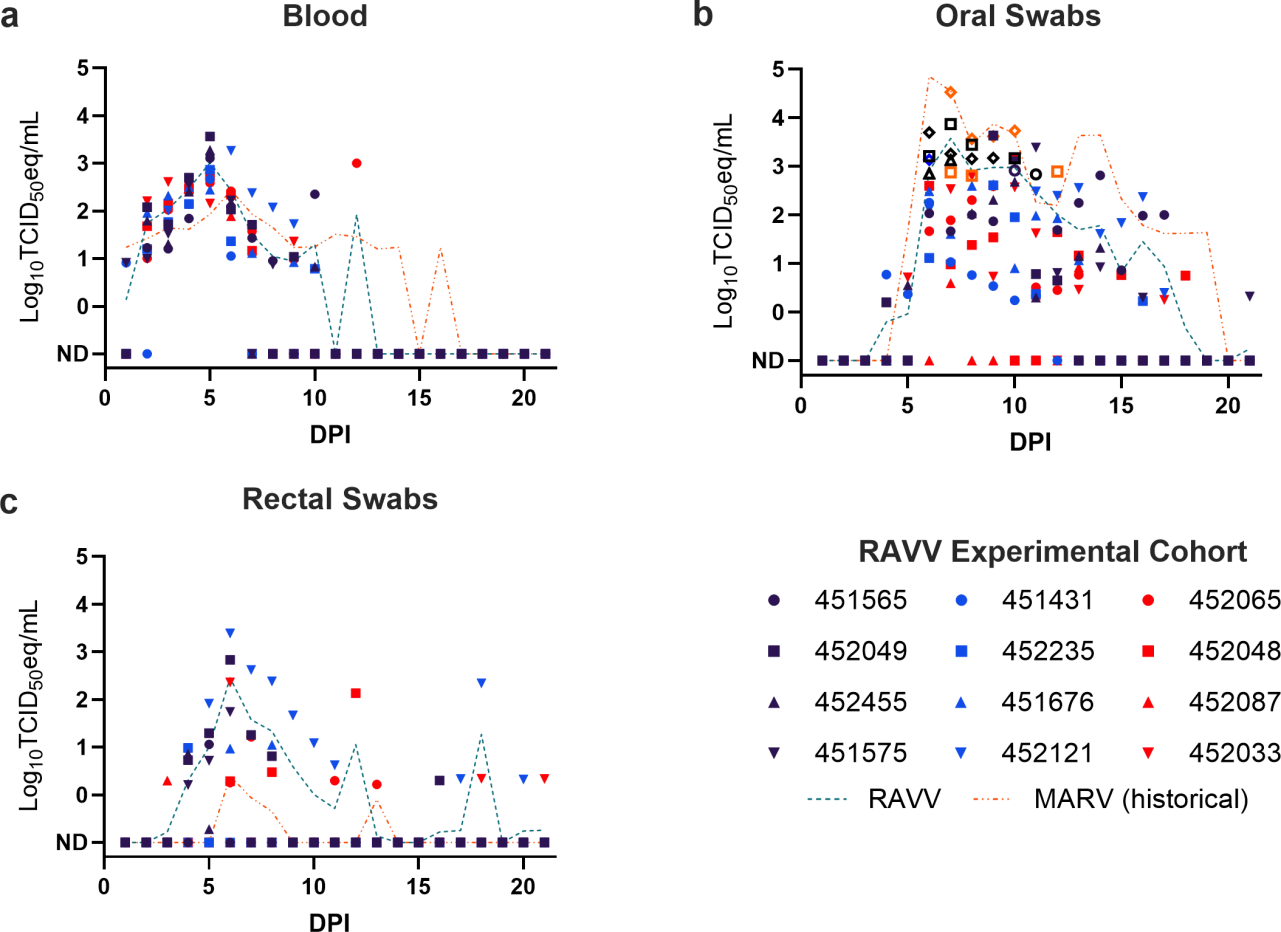
RAVV shedding dynamics in experimentally infected Egyptian rousette bats. RAVV loads (RT-qPCR-derived log_10_TCID_50_ equivalents/mL) in (a) blood, (b) oral swabs, and (c) rectal swabs from experimentally infected Egyptian rousette bats. The teal dashed line represents the overall mean RAVV load in each sample. The orange dashed line represents the overall mean MARV load taken from historical MARV data (14). Open symbols in b represent oral swabs from which infectious RAVV was isolated. ND: not detected.

**Fig 3:**
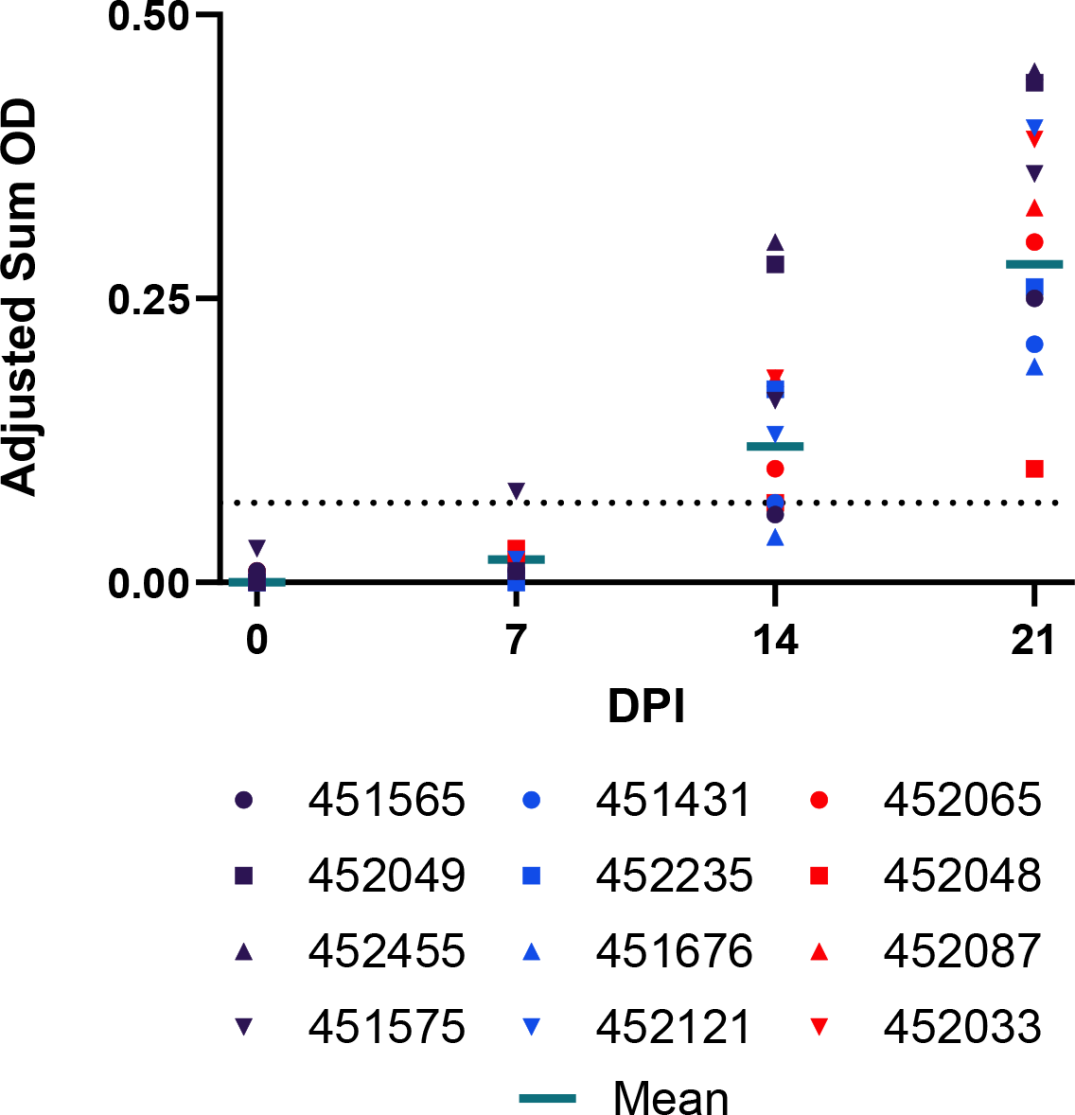
RAVV IgG antibody responses of experimentally infected Egyptian rousette bats. IgG antibodies were detected by ELISA with purified recombinant nucleoprotein of the Angola strain of MARV expressed in *Escherichia coli* from blood taken at 0, 7, 14, and 21 DPI. IgG antibody levels are expressed as adjusted sum OD values normalized between 0 and 1. The black dotted line represents the assay threshold (RAVV seropositive ≥ 0.07).

Prior to inoculation, none of the bats had detectable viremias (Fig. 2a) or anti-RAVV IgG (Fig. 3), indicating no prior exposure to RAVV. Viral RNA levels in blood (Fig. 2a) quantified through RT-qPCR analysis of viral RNA and presented as mean TCID_50_ equivalents per milliliter of fluid were consistent with previous studies that characterized MARV infection and shedding dynamics (14, 21). RAVV viremia was detected in all 12 ERBs, with mean viral load values peaking on Day 5 (mean: 1.03 × 10^3^ TCID_50_/mL; highest individual value: 3.65 × 10^3^ TCID_50_/mL) and cleared by Day 13. The highest number of viremic ERBs was on 3–6 DPI (*n* = 12 bats each day), with 79 positive viremic data points overall. The average length of detectable viremia was 6.6 days, ranging from 5 to 8 days. Blood samples for RT-qPCR were no longer collected after 15 DPI following three consecutive days of negative RT-qPCR results from all bats.

RAVV RT-qPCR positive oral swabs were detected in all 12 ERBs, with mean viral load values peaking on 7 DPI (mean: 3.76 × 10^3^ TCID_50_/mL; highest individual value: 3.33 × 10^4^ TCID_50_/mL) and with sporadic RT-qPCR positivity through study completion at 21 DPI. The highest number of positive oral swab specimens was 7 DPI (*n* = 12), with 106 positive oral swab specimens overall. The average length of oral shedding was 8.8 days, ranging from 4 to 12 days. RT-qPCR-positive oral swab specimens with CT values ≤ 32 were selected for virus isolation; this represented 26 out of the 106 (24.5%) RAVV RNA positive oral swab samples from eight infected ERBs (451565, *n* = 3 samples; 451575, *n* = 6; 452033, *n* = 2; 452049, *n* = 5; 452065, *n* = 1; 452121, *n* = 4; 452235, *n* = 3; 452455, *n* = 2). Infectious RAVV was isolated from 20/26 (77%) samples.

RAVV RT-qPCR-positive rectal swabs were detected in 11/12 (92%) ERBs, with mean viral load values peaking on 6 DPI (mean: 2.87 × 10^2^ TCID_50_/mL; highest individual value: 2.46 × 10^3^ TCID_50_/mL) and with sporadic RT-qPCR positivity through study completion at 21 DPI. Bat 451431 never had a RAVV-RT-qPCR-positive rectal swab despite having positive oral swab and blood samples. The highest number of positive rectal swab specimens was at 6 DPI (*n* = 7), with 36 positive rectal swab specimens overall. The average length of rectal shedding was 3 days, ranging from 1 to 10 days. To avoid mucosal irritation or injury, duplicate rectal swab samples were not collected for isolation; therefore, isolation attempts on rectal swab samples were not performed.

### Complete seroconversion in all bats

Consistent with previous studies (14, 19–21, 45), all ERBs demonstrated a robust primary immune response, with all inoculated bats (12/12) testing RAVV seronegative at 0 DPI and subsequently seroconverting to RAVV [mean peak adjusted sum optical density (OD) = 0.28, s.d. = 0.11; Fig. 3] by 21 DPI.

### Heterogeneities in oral and rectal RAVV shedding

**Fig 4:**
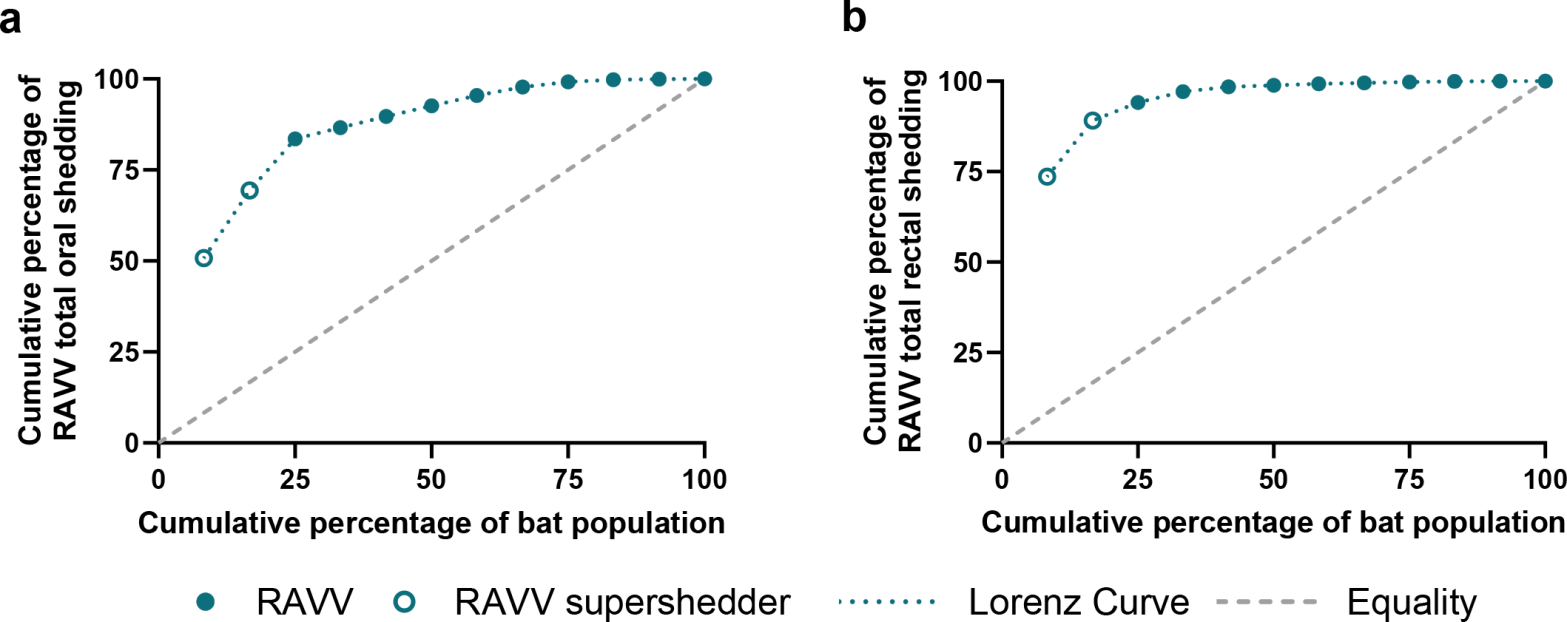
Cumulative RAVV shedding in experimentally infected Egyptian rousette bats. Lorenz curve of cumulative percentage of the inoculated bat population versus cumulative percentage of (a) oral and (b) rectal shedding ranked in descending order (i.e., the first circle on the circle represents bat 452121, which had the highest cumulative percentage of both rectal and oral shedding).

Heterogeneity in host shedding of pathogens plays an important role in infectious disease transmission dynamics and can be measured by assessing cumulative pathogen loads shed in excretory products of naturally or experimentally infected individuals (46–57). Viral shedding was calculated for each inoculated bat by summing RAVV RNA loads detected 0–21 DPI in oral and rectal swabs. Total oral and rectal shedding varied considerably between individual bats, with sum log_10_TCID_50_ equivalents/mL ranging from 1.21 to 4.68 (mean = 3.29, s.d. = 0.91) and undetectable to 3.54 (mean = 1.56, s.d. = 1.03), respectively. As previously demonstrated (14), the Lorenz curve and associated Gini coefficient are effective at illustrating and quantifying inequality in a distribution and herein used to highlight heterogeneity in individual RAVV oral (Fig. 4a) and rectal (Fig. 4b) shedding. For example, Fig. 4a demonstrates that 25.0% of the inoculated bats were responsible for 83.5% of RAVV oral shedding; 50.0% of the bats were responsible for 92.6% of oral shedding; and 75.0% of the bats were responsible for 99.2% of oral shedding. Figure 4b demonstrates that 25.0% of the inoculated bat population was responsible for 94.1% of RAVV rectal shedding; 50.0% of the bats were responsible for 98.9% of rectal shedding; and 75.0% of the bats were responsible for 99.8% of rectal shedding. Using a previously established approach (14, 57), two inoculated bats (452121 and 452049) were classified as supershedders for both oral and rectal RAVV shedding, as both shed at levels greater than the 80th percentile (oral = 4.18 log_10_TCID_50_ equivalents/mL; rectal = 2.64 log_10_TCID_50_ equivalents/mL, respectively) and together accounted for 69.4 and 89.2% of the total RAVV oral and rectal shedding, respectively. RAVV oral and rectal shedding was detected 12 and 10 times in bat 452121 and eight and six times in bat 452049, respectively. An infectious virus was isolated from four out of four (100%) oral swabs taken from bat 452121 and four out of five (80%) oral swabs taken from bat 452049.

### No evidence of clinical disease

**Fig 5:**
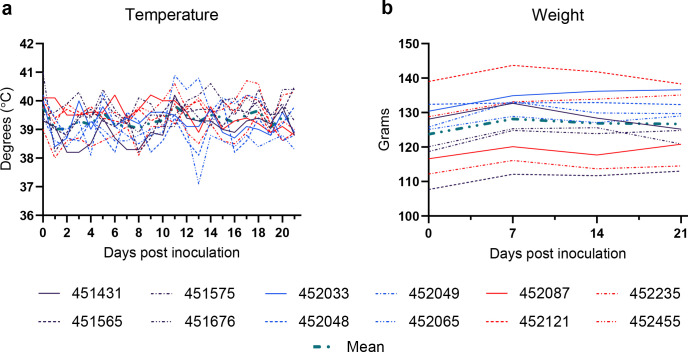
Clinical data. (a) Temperatures (°C) acquired via rectal thermometer and (b) weights (g) from experimentally infected Egyptian rousette bats.

Consistent with previous MARV experimental studies in ERBs (14, 21), no disease-related morbidity or mortality was observed in any of the ERBs, and normal social and feeding behaviors were maintained. All bats maintained normal body weights and rectal temperatures, consistent with previous studies (Fig. 5) (21).

## DISCUSSION

This study provides the first measure of the shedding dynamics of experimental RAVV infection in ERBs, a natural reservoir host. Similar to MARV, RAVV reaches a viremic peak at approximately 5–6 DPI, with viral shedding peaking in oral secretions around 7 DPI and in fecal secretions around 6 DPI (14, 18, 21). These findings suggest that RAVV, like MARV, is likely horizontally transmitted through direct and/or indirect contact with infected bodily fluids (14, 21). A recent study found that infectious MARV can persist on contaminated fruit spats for up to 6 h, providing an additional route of exposure to other animals, including other bat species or other susceptible animal hosts, such as non-human primates (58).

A comparison between RAVV and MARV shedding dynamics can be drawn between the current study and that of Schuh et al. (14). In Schuh et al. (14), 12 naïve ERBs were subcutaneously inoculated with an identical experimental dose (four log_10_TCID_50_) and route of MARV and underwent oral and rectal swab sampling over an identical timeline to the current RAVV study. While the infection timeline between MARV and RAVV is comparable, experimentally inoculated ERBs produce higher rectal RAVV shedding loads with a longer shedding duration compared to Schuh et al. (14), along with prolonged oral shedding and higher peak viremia (Fig. 3). This striking variation in rectal shedding may elucidate why, in a recent surveillance study of rectal swab samples from ERBs in South Africa, only RAVV was detected out of 416 samples tested (8).

It is currently unclear as to how these two orthomarburgviruses, RAVV and MARV, continue to circulate in free-ranging ERB populations yet remain genetically distinct. As mentioned earlier, the full genomic sequence of RAVV differs by up to 21% from MARV (Fig. 1), with the most notable variation (~22%) in the amino acid sequence of the RAVV glycoprotein (GP) (6, 16). Investigation into the generation of protective and cross-reactive monoclonal antibodies via exposure to engineered MARV GPs found that RAVV has four unique residues on the GP2 “wing,” which is a 66-amino-acid N-terminal GP2 extension, when compared to other MARV variants (59). Additionally, a recent study investigating the use of mRNA vaccines against MARV and RAVV developed based on sequences of their respective GPs and glycan caps found differences in antibody frequencies, antibody binding, neutralizing capacity, and linear epitope recognition (60). This suggests that the structural divergence between the GPs of distant orthomarburgviruses potentially affects stability, alterations, and/or the spatial location of domains (60). These differences may contribute to the differences noted between RAVV and MARV shedding in this study.

Variations in viral GPs of other viruses can have significant implications for infection and pathogenicity (61). For instance, a point mutation in the influenza C virus GP was found to increase receptor-binding efficiency (62). Similarly, mutations within the hepatitis C virus E2 GP increased its affinity to its receptor and reduced the virus’ sensitivity to neutralization (63). An amino acid mutation in the GP of lymphocytic choriomeningitis virus (Clone 13 strain) is thought to be responsible for the long-term persistence of Clone 13 infections (64). Additionally, six mutations generated *in vitro* at the interface of Ebola virus (EBOV) GP_1_ and GP_2_ resulted in confirmational changes that rendered the virus independent of Cathepsin B, a protease required for EBOV cellular entry (65). Substitutions in many conserved residues of the MARV GP led to significant defects in GP expression, incorporation into HIV virions, and the ability to mediate viral entry (66). Furthermore, a naturally occurring polymorphism in the Sudan virus GP_1_ decreased GP stability, therefore potentially affecting viral infectivity (67). These findings underscore the critical role of glycoprotein variations in viral infectivity, stability, and immune evasion, highlighting their potential as targets for therapeutic intervention and possibly explaining the variation in fecal shedding dynamics observed thus far in ERBs experimentally infected with either RAVV or MARV.

Variation in infection and shedding loads and their impact on disease transmission dynamics have been well documented in both human (49, 52, 68) and veterinary literature (14, 46, 48, 50, 57, 69–71) and linked to between- and within-host variations (53, 55, 69, 72), immune suppression (68), and viral (68, 73), bacterial (74–76), and/or parasitic co-infections (75). The Pareto Principle (or 80/20 rule) originally applied to wealth inequality states that ~80% of an effect (i.e., RAVV virus shedding) is produced by ~20% of the population (56, 57). This principle has been previously applied to experimental ERB MARV infection dynamics (14), where the Lorenz curve and the Gini coefficient are used to graphically represent and quantify the cumulative inequality in viral shedding to allow for identification of viral “supershedders” (57) within the experimental cohort. Super-spreaders, or supershedders, are individuals who infect or shed disproportionately more than most infected individuals and have been described in both natural (57, 69, 77) and experimental infections (14), in which the infectious exposure dose, time course, and inoculation route are standardized. In this study, we found that 25.0% of the bats were responsible for 83.5% of RAVV oral shedding, and 16.7% of the bats were responsible for 89.2% of the RAVV rectal shedding. Two bats (452121 and 452049) were classified as supershedders (57) for both oral and rectal RAVV shedding, as both shed at levels greater than the 80th percentile and accounted for 69.4 and 89.2% of the total RAVV oral and rectal shedding, respectively. Both supershedder bats also had prolonged shedding durations: bat 452121 shed RAVV orally for 12 days and rectally for 10 days, while bat 452049 shed RAVV orally for 8 days and rectally for 6 days. MARV loads detected in rectal swabs in Schuh et al. (14) were excluded from cumulative shedding calculations, making a direct comparison between MARV and RAVV cumulative rectal shedding unavailable. This heterogeneity in RAVV shedding loads and durations may contribute to the fitness of RAVV in natural outbred ERB populations. Additionally, the marked statistical significance in RAVV rectal shedding compared to MARV rectal shedding introduces the possibility that rectal shedding is a more robust component of environmental maintenance and spread for RAVV than it is for MARV.

Another factor that cannot be discounted in the variation in viral shedding dynamics between the current study and previous ERB MARV experimental studies could be age. Ecological studies have observed a seasonal pulse in viral circulation within natural bat reservoir hosts, with a higher prevalence of active infection in juvenile bats (~6 months old) (10, 78–80) and age-dependent variations in disease outcomes (81, 82). This may reflect a “perfect storm” of reservoir infective permissiveness, in which the weaned juvenile bats are no longer protected by material antibodies, are susceptible to horizontal transmission of infectious disease within the densely packed roosts, and lack immunologic maturity to temper viral infection and subsequent shedding. As such, the standard experimental model for most MARV ERB research has been to use animals that are 5–7 months old to mimic the biological parameters found in free-ranging ERBs. Here, the ERBs in the current study were 12–14 months old, introducing the possibility that age-related variation in infection dynamics could be a factor in the observed experimental differences in viral rectal shedding.

This study shows that experimental RAVV infection in ERBs, a natural reservoir host for *Orthomarburgvirus marburgense* viruses, follows a similar viral shedding timeline as past experimental MARV infections in ERBs but with increased virus rectal shedding. Future work is needed to fully characterize the pathogenesis of experimental RAVV infection in ERBs, including evaluation of clinical, histochemical, and immunohistochemical findings and tissue viral loads at serial time points, with heightened focus on the small and large intestines. Future RAVV research could additionally include comparisons to Sosuga virus, a paramyxovirus for which the ERB serves as a putative natural reservoir, as Sosuga virus has been shown to replicate extensively in the small intestines (83). This work provides an important baseline for hypothesis-driven research, allowing a successful extrapolation of research findings in controlled laboratory settings to wild ERB populations and enabling experimental comparisons between MARV and RAVV infections.

## MATERIALS AND METHODS

### Animals and biosafety

All experimental procedures were conducted with approval from the Centers for Disease Control and Prevention (CDC, Atlanta, Georgia, USA) Institutional Animal Care and Use Committee and in strict accordance with the Guide for the Care and Use of Laboratory Animals (Committee for the Update of the Guide for the Care and Use of Laboratory Animals 2011). The CDC is a fully accredited research facility by the Association for Assessment and Accreditation of Laboratory Animal Care International. No recombinant or human patient-derived clinical materials were used in these studies.

Procedures conducted with infectious RAVV or infected bats were performed at the CDC under biosafety level 4 (BSL-4) laboratory conditions in accordance with select agent regulations (Animal and Plant Health Inspection Service and Centers for Disease Control and Prevention 2014). All investigators and animal handlers followed strict BSL-4 safety and infection control practices (84).

A total of 12 adult captive-born (21) ERBs (12–14 months old; six males and six females) were used in this study. The bats were housed in groups of six separated by sex in designated experimental and control caging (interior dimensions 61 × 71 × 76 cm long, wide, and high, respectively) in a climate-controlled BSL-4 laboratory animal room with a 12 h day/night cycle. The cages were housed within an isolation unit (Duo-Flow Mobile Units, Lab Products, Inc., Seaford, Delaware, USA) with high-efficiency particulate air-filtered inlet and exhaust air supply. The bats’ daily food consisted of chopped bananas, watermelon, cantaloupe, seedless grapes, apples, and pears dusted with a protein vitamin supplement (Lubee Bat Conservancy, Gainesville, Florida, USA).

### Virus

Following the experimental design of a previous MARV study (14), 4 log_10_ 50% tissue culture infective dose (TCID_50_) of a RAVV isolate (188bat2007 virus; second passage on Vero E6 cells) obtained from a naturally infected ERB (188bat) collected during a 2007 orthomarburgvirus outbreak ecological investigation at Kitaka Mine in southwestern Uganda was prepared in 0.25 mL of sterile Dulbecco’s modified Eagle’s medium (6).

### Experimental design

ERBs were acclimated in the BSL-4 laboratory for 7 days before the beginning of the study (acclimation phase). Baseline blood samples, body weights, and temperatures were recorded prior to inoculation. At 0 DPI, all 12 bats were inoculated subcutaneously under isoflurane anesthesia with the above-described RAVV inoculum in the caudal abdominal region. As data from historical MARV control ERBs from previous studies were available, control ERBs were not utilized in this study. Blood samples, oral swabs, rectal samples, and temperatures were recorded daily; body weight was measured on 0, 7, 14, and 21 DPI.

### Specimen collection

Specimen collection has previously been described in detail (14, 18, 21). Blood (whole, nonheparinized; 10 and 21 µL for RT-qPCR and serology, respectively) was taken on −1 DPI and daily from 1 to 21 DPI from the cephalic wing vein using a sterile lancet (C&A Scientific, Manassas, VA, USA). Blood was tested for the presence of RAVV RNA by RT-qPCR through 15 DPI, and RAVV IgG antibody responses were monitored weekly through 21 DPI. The oral mucosa was sampled daily through 21 DPI by swabbing the inside of the bat’s mouth and cheeks using two polyester-tipped applicators (Fisher Scientific, Grand Island, NY, USA). After sampling, one oral swab was immediately placed in either a deep-well plate with 500 µL of MagMAX lysis buffer solution (Life Technologies, Grand Island, New York, USA) for RT-qPCR analysis, and one oral swab was placed in sterile viral transport medium for attempted virus isolation of any RAVV RNA positive swabs. A temperature probe covered with a plastic sheath (MABIS Healthcare, Waukegan, Illinois, USA) was used to measure the daily rectal temperature of each bat. The plastic sheath was then cut and placed into a deep-well plate with 500 µL of MagMAX lysis buffer solution (Life Technologies) for fecal RT-qPCR analysis.

### Euthanasia

At 22 DPI, all bats were euthanized under anesthesia via an overdose of isoflurane, followed by cardiac exsanguination. Cardiac blood was collected and retained.

### Nucleic acid extraction

Nucleic acid was extracted from blood, oral swab, and rectal probe covers using the MagMAX Pathogen RNA/DNA Kit (Thermo Fisher Scientific, Waltham, MA, USA) on the MagMAX Express-96 Deep-well Magnetic Particle Processor (Thermo Fisher Scientific).

### RT-qPCR

RT-qPCR procedures have been previously described in detail (14, 18, 21). Reverse-transcribed RAVV and ERB beta-2-microglobulin (B2M) RNA were detected on the CFX Opus 96 Real-time PCR System (Bio-Rad, Hercules, CA, USA) using the Luna Probe One-step RT-qPCR 4× Mix with UDG (New England Biolabs, Inc., Ipswich, MA, USA). The amplification utilized primers and reporter probes targeting the orthomarburgvirus viral protein 40 (VP40) gene (forward primer: GGACCACTGCTGGCCATATC, reverse primer: GAGAACATITCGGCAGGAAG, probe 1: 56-*FAM*-ATC CTA AAC-*ZEN*-AGG CTT GTC TTC TCT GGG ACT T-*3IABkFQ*, probe 2: 56-*FAM*-ATC CTG AAT-*ZEN*-AAG CTC GTC TTC TCT GGG ACT T-*3IABkFQ*) and the ERB B2M gene (forward primer: CAGCAAGGACTGGTCTTTCTAT, reverse primer: CCTCCATGATGCTGGTTAGTT, probe: *FAM*-TTC ACA CGG-*ZEN*-CAG CTG TAC TCA TCC-3*IABkFQ*), respectively. This assay was designed to detect a conserved sequence of VP40 present in all known species of orthomarburgvirus, including RAVV (16). Relative RAVV TCID_50_eq/mL (blood and oral specimens) were interpolated from standard curves generated from serial dilutions of the titrated 188bat RAVV spiked into appropriate biological specimens. Based on testing triplicate 10-fold serial dilutions of RAVV ranging from 5.8 × 10^6^ to −1.12 × 10^6^ TCID_50_eq/mL, the lowest concentration of RAVV detected in all three replicates was 1.8 × 10^6^ TCID_50_eq/mL.

### Virus isolation and immunofluorescence assay

Virus isolation and immunofluorescence assays have been previously described in detail (14, 18, 21). Virus isolation was attempted on RT-qPCR-positive oral swab samples for orthomarburgvirus with CT values ≤ 32. Initially, monolayers of 85% confluent Vero E6 cells (American Type Culture Collection, CRL-1586) in 25 cm^2^ tissue culture flasks were inoculated with 100 µL viral transport medium from wells containing the positive oral swabs supplemented with 500 µL maintenance media (Dulbecco’s Modified Eagle Medium containing 2%, heat-inactivated fetal bovine serum, 100 units/mL penicillin, 100 µg/mL streptomycin, and 2.50 µg/mL amphotericin B) and incubated for 1 h at 37°C/5% CO_2_. Subsequently, 7 mL of maintenance media was added, and cultures were further incubated under the same conditions. At 7 and 14 DPI, tissue culture monolayers were scraped to release virus-infected cells. Next, 1.5 mL of each cellular medium was suspended in 8 mL borate saline. After centrifugation to pellet the cellular suspensions, borate saline was decanted, and the cells were resuspended in 500 µL borate saline. Then, 25 µL of the cellular resuspensions was spotted onto 12-well spot slides, which were fixed in acetone and exposed to 2 megarads of γ-irradiation.

All 7 and 14 DPI cultures were tested by immunofluorescence assay for orthomarburgvirus antigen. Spot slides were incubated with a 1:100 dilution of rabbit anti-MARV polyclonal (in-house) or normal rabbit serum (negative control; in-house) for 30 min at 37°C. Slides were then rinsed twice with 1× PBS for 10 min, followed by incubation with 1:40 dilution of goat anti-rabbit fluorescein isothiocyanate (Capel-ICN Pharmaceuticals, Aurora, OH, USA) for 30 min at 37°C. After a 7 min rinse with 1× PBS, the slides were stained with Eriochrome Black T (in-house) for 7 min, followed by another 7 min rinse with 1× PBS. The slides were then observed under a fluorescence microscope.

### Serology

As previously described (14, 21, 22), ELISA plates were coated with 50 ng per well of purified recombinant Marburg Angola NP or Reston NP expressed in *Escherichia coli* (GenScript, Piscataway, NJ, USA) diluted in PBS containing 1% thimerosal. Following an overnight incubation at 4°C, the plates were washed with PBS containing 0.1% Tween-20 (PBS-T). A 1:100 dilution of gamma-irradiated bat whole blood in masterplate diluent (PBS containing 5% skim milk powder, 0.5% Tween-20, and 1% thimersol) was added to the first well, with subsequent fourfold serial dilutions in serum diluent (PBS containing 5% skim milk and 0.1% Tween-20) performed through 1:6,400. After incubating for 1 h at 37°C, the plates were washed with PBS-T, and bound antibodies were detected using a 1:11,000 dilution of anti-goat bat IgG (Bethyl Laboratories, Montgomery, TX, USA) in serum diluent. Following a 1 h incubation with the secondary antibody at 37°C, the plates were washed twice with PBS-T, and the 2-Component ABTS Peroxidase System (KPL, Gaithersburg, MD, USA) was added. The substrate was allowed to incubate for 30 min at 37°C before the plates were read on a microplate spectrophotometer at 410 nm.

To negate non-specific background reactivity, adjusted optical density (OD) values were calculated by subtracting the ODs at each fourfold dilution of wells coated with Reston NP from ODs at corresponding wells coated with MARV NP. The adjusted sum OD value was then linearly transformed using the min–max normalization method. The seropositivity threshold was set at 0.07 after in-house assay optimization.

### Data and statistical analyses

Statistical analyses were completed as previously reported (14). Each comparator group comprised 12 bats. The number of bats per group was based on the reproductive capacity of the ERB breeding colony, the number of bats that could be safely handled daily, and the available space in the BSL-4 lab. Investigators were not blinded during the study, and no bats or individual data points were excluded from the analyses.

Excel (Microsoft 365, Redmond, WA) was used to manage data, and GraphPad Prism 10 (GraphPad, La Jolla, CA) was used to perform statistical analyses and generate figures. The RAVV peak viral loads and the duration of viral shedding were determined for each bat according to the sample type (blood, oral swab, and rectal swab). To assess RAVV infectiousness, cumulative viral shedding loads were calculated for each bat by summing the viral loads detected in blood, oral swabs, and rectal swabs through the duration of the study. Using the approach of Jankowski et al. (57), bats were classified as supershedders if they shed RAVV at loads ≥ the 80th percentile. Raw data from Schuh et al. (14) were generously provided by the author for MARV statistical comparison.

The Shapiro–Wilk test was used to determine if the peak viral load, duration of viral shedding, and cumulative viral shedding load data sets followed a normal or lognormal distribution. If data sets were normally distributed, then unpaired *t*-tests were used to determine if parameter means differed significantly between RAVV and MARV bat groups. If data sets were lognormally distributed, they were log-transformed before using unpaired *t*-tests to determine if parameter geometric means differed significantly between RAVV and MARV bat groups. If data sets did not follow a normal or lognormal distribution, then non-parametric Mann–Whitney *U* tests were used to determine if the parameter mean ranks differed significantly between RAVV and MARV bat groups. All *P* values are two-tailed, and *P* < 0.05 is considered statistically significant. Each bat represents an individual biological replicate.

## Data Availability

All data generated or analyzed during this study are included in this published article or are available upon request from the corresponding author.
